# The Internal Chicken-mite (Cytodites Nudus Vizioli)

**Published:** 1898-08

**Authors:** E. V. Wilcox

**Affiliations:** Montana Experiment Station


### THE INTERNAL CHICKEN-MITE (CYTODITES NUDUS VIZIOLI.)

By E. V. Wilcox,
MONTANA EXPERIMENT STATION.

Since 1858, when Gerlach studied this mite, it has received attention from various writers. It has been found in large numbers in the lungs, bronchial tubes, air-sacs, hollow bones, and upon the peritoneum of gallinaceous birds.

On several points opinions have varied to a considerable extent among the investigators of cytodites. At first the question of the relationship of the mite furnished some difficulty. It has not been discovered how the mite is transmitted from fowl to fowl, or by what course it gets into the air-sacs and upon the peritoneum of the fowl. Furthermore, it is as yet by no means determined that cytodites is of great economic importance.

The latest paper on the subject which has come to the notice of the writer is by W. L. Williams, in the *American Veterinary Review* for April, 1898. The paper is so misleading in its general tendency, so exaggerated in its statements, and so inconsistent in its logic, that a brief criticism seems necessary.

The author states that he has found no record of the occurrence of the mite in any English-speaking country. Apparently he has not searched the literature very thoroughly. In Osborn's Bulletin on "Insects Affecting Domestic Animals," 1896, page 263, is a record of the occurrence of the mite in Baltimore, and, as Osborn adds, it had already been reported in Washington by Riley in the *American Naturalist*, vol. xvii. p. 422.

In regard to the economic importance of this mite, we believe that Williams' statements are much exaggerated. His work on the subject was done in Montana, and the author asserts that poultry-raising in Montana is "generally unprofitable," and that "the most serious and persistent malady of poultry was that due to the air-sac mite."

For a period of two years, in the same locality where Williams asserts that the poultry business is unprofitable on account of the great depredations of cytodites, we have taken every opportunity to kill and examine diseased fowls. We found cytodites in five of these fowls.

Moreover, on looking for the facts upon which Williams' statements are based, we find that he has not produced the slightest evidence of any injury or lesion which is due to the mites. The author acknowledges that his statements are mere conjectures, when he says: "Although I am unable to define the manner, I am nevertheless thoroughly convinced with Gerlach and Zundel that they do produce disease and death."

One author has tried experimental transmission of the mites into healthy fowls, both through the larynx and directly through the body-wall into the air-sacs. Of one such experiment the author relates: "No symptoms of disease developed, and after an interval of forty-seven days the chick was killed by bleeding, and the autopsy revealed a few cytodites in varying stages of development, including young mites and pregnant females. No trace of pathological lesions was discovered."

But, although all his experiments gave negative results, and although his autopsies revealed no lesions attributable to the mites, the author is " nevertheless thoroughly convinced " that the mites are, perhaps, the most destructive enemy of poultry. A scientific worker should be able to give a reason for the faith that is in him.

We are not prepared to assert that cytodites is harmless. We do maintain, however, that absolutely no evidence has been presented by Williams of any lesion or disease for which cytodites is responsible. He is, therefore, not justified in speaking of a " malady " or " disease in this connection." Williams speaks of " importing and cultivating the disease." He may have imported and cultivated mites, but, according to his own account, he was unable to produce any " disease " by the artificial transmission of the mites. Williams, moreover, is unable to define any disease caused by cytodites. There was no constant set of symptoms in his subjects, and there were absolutely no pathological lesions by which any disease could be described.

The article under discussion is one more example of the result of hastening into print on a supposedly new subject without considering the question whether or not one has any results to publish. No contribution to our knowledge of cytodites is made in the article. It is only a rehearsal of what has been written by Gerlach, Zurn, Zundel, Holzendorff, Vizioli, and especially Megnin. Indeed, the whole description of the genus and species, of the anatomical peculiarities, of the ovigerous female, male, octopod, larva, etc., occupying nearly five pages of the article, is simply an adaptation from Megnin. Megnin's order of treatment has been followed in detail, even in the order of words. A large part of the description is a tolerably close translation of Megnin, and should, according to our notion of scientific etiquette, have been included in quotation marks and accredited to Megnin.

It is rather amusing to find, at the end of Williams' translation of Megnin, the statement that " these observations, in connection with the anatomical characters of the parasite, fully support Megnin," etc. It would seem that one's own words, when translated into another language, may be used in " support " of the original statement.

We select, as an illustration, a passage from Megnin and a corresponding passage from Williams. The article by Megnin to which we refer is in the *Journal de l'Anatomie et de la Physiologie*, 1897, pp. 123–153. On page 151 we read : " Jeune femelle pubere semblable au male, s'en distinguant par l'absence complete d'organes genitaux. C'est elle qui s'accouple avec le male, et la copulation se fait par l'anus, comme chez l'immense majorite des Acarieus.''

Compare with this the following from page 14 of Williams' article: "The young non-gravid female is distinguished from the male by the absence of genitals, copulation being effected through the anus as in most acaria." Other parallel statements could be cited. On the page following the above quotation from Williams we find the statement that he has "failed to observe the copulatory process." Such curious inconsistency would not be possible if the whole article were written from personal observations. We should hesitate to describe a process which we had never seen.

The drawings accompanying the article are done by Mrs. Comstock, and are very accurate.

